# HHV‐8‐Associated Hemophagocytic Lymphohistiocytosis in a HIV‐Negative and Nontransplant Man: A Case Report and Literature Review

**DOI:** 10.1155/crdi/9882834

**Published:** 2025-12-19

**Authors:** Yu Wang, Jun Hu, Feng Zhu, Lu Cheng

**Affiliations:** ^1^ Department of Pediatrics, Children’s Hospital of Zhejiang University School of Medicine, Hangzhou, Zhejiang, China, zju.edu.cn; ^2^ Department of Surgical Intensive Care Unit, Second Affiliated Hospital of Zhejiang University, Hangzhou, Zhejiang, China; ^3^ Department of Hematology, Children’s Hospital of Zhejiang University School of Medicine, Hangzhou, Zhejiang, China, zju.edu.cn; ^4^ Department of Pathology, Second Affiliated Hospital of Zhejiang University, Hangzhou, Zhejiang, China

## Abstract

Hemophagocytic lymphohistiocytosis (HLH) is a critical and life‐threatening syndrome, which can rapidly progress to mortality without immediate or targeted therapeutic intervention. Human herpesvirus 8 (HHV‐8, or Kaposi sarcoma–associated herpesvirus (KSHV)) is a rarely described viral cause of secondary HLH, especially in immunocompetent adults. Here, we report an uncommon case of HLH after HHV‐8 infection, with no history of transplantation or HIV infection. The pathophysiological mechanisms of secondary HLH remain incompletely understood. There might be potential overlapping pathogenic pathways between HHV‐8‐associated HLH and KICS pathogenesis. E3 ubiquitin ligases may be a critical factor in the pathogenesis of HHV‐8‐associated secondary HLH. Ferritin testing, peripheral blood smears, and pathogenesis evaluations should be immediately implemented for patients presenting with fever and bicytopenia. Through our case report, we seek to advance clinical recognition of HHV‐8‐associated HLH, optimize diagnostic precision, and ultimately improve survival outcomes for patients.

## 1. Introduction

Hemophagocytic lymphohistiocytosis (HLH) is a critical and life‐threatening syndrome, which can rapidly progress to mortality without immediate, targeted therapeutic intervention. HLH is classified as primary HLH and secondary HLH. The prevalence of secondary HLH remains undetermined due to substantial underrecognition and diagnostic challenges in identifying potential cases [1]. In England, the incidence rates of HLH increased from 1.06 cases per one million in 2003 to 4.22 cases per one million in 2018 [2]. Chronic autoimmune comorbidities were documented in 92% of HLH‐diagnosed patients [2]. The pathophysiological mechanisms of secondary HLH remain incompletely understood. The prognosis of HLH is poor, especially for those patients without appropriate treatment. Human herpesvirus 8 (HHV‐8, or Kaposi sarcoma–associated herpesvirus (KSHV)) was discovered to be the cause of Kaposi sarcoma [3]. HHV‐8 is the etiologic agent of other diseases associated with human immunodeficiency virus (HIV) infection, such as primary effusion lymphoma (PEL), multicentric Castleman disease (MCD), and Kaposi sarcoma inflammatory cytokine syndrome (KICS) [4]. HHV‐8 is a rarely described viral cause of secondary HLH, especially in immunocompetent adults. Herein, we report an uncommon case of HLH after HHV‐8 infection. A 79‐year‐old man with no history of transplantation or HIV infection presented fever, splenomegaly, bicytopenia (hemoglobin and platelets), and hyperferritinemia. In addition, the plasma metagenomic next‐generation sequencing (mNGS) showed a high HHV‐8 viral load. Further examination (hemophagocytosis and elevated soluble CD‐25 level) confirmed a diagnosis of HLH. We report this case to remind clinicians to consider the possibility of secondary HLH after HHV‐8 infection and provide a starting point to discuss mechanisms of HHV‐8‐associated secondary HLH.

## 2. Case Report

**Figure 1:**
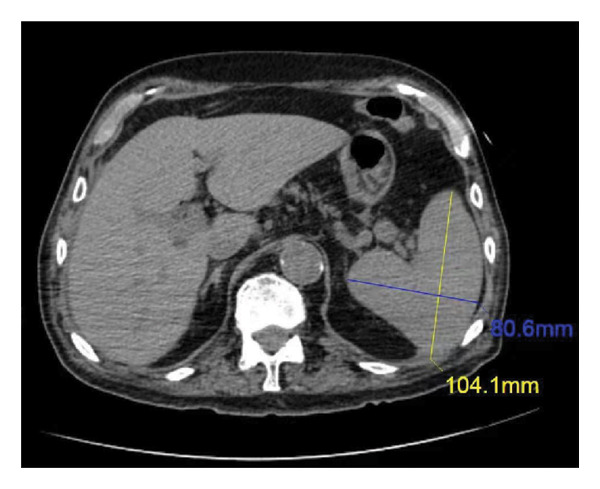
Results of abdominal CT on admission. Abdominal CT showing mild splenomegaly (104.1∗80.6 mm) with an intact capsule and minimal perihepatic and perisplenic fluid.

A 79‐year‐old man was admitted to the hospital with appetite reduction for 25 days and fever for 10 days. The patient presented undiagnosed fever fluctuates between 38.3°C and 39.3°C. Physical examination of the patient did not reveal any pathological finding. The patient had a history of chronic obstructive pulmonary disease for several years. He had been taking appropriate oral glucocorticoids intermittently over the past 6 months. The patient had no history of any transplantation or HIV infection. The laboratory data were listed in Table 1. The patient presented anemia, thrombocytopenia, and hyperferritinemia. Abdominal ultrasound and CT scan showed mild splenomegaly without lymphadenopathy (Figure 1). To clarify the diagnosis and refine the differential diagnosis, bone marrow biopsy and peripheral blood smear were conducted.

**Table 1:** Laboratory data.

	On admission	References range
White cell count (× 10^9^/L)	4.9	4.0–10.0
Neutrophils count (%)	68.8	50.0–70.0
Lymphocytes count (%)	11.5	20.0–40.0
Monocytes count (%)	19.4	4.0–12.0
Eosinophils count (%)	0.1	0–10.0
Hemoglobin (g/L)	85	131–172
Platelet count (× 10^9^/L)	60	100–300
Reticulocyte count (%)	6.61	0.58–1.59
C‐reactive protein (mg/L)	88.6	< 10.0
Procalcitonin (ng/mL)	1.36	< 0.5
Creatinine (μmol/L)	117	57–115
Total bilirubin (μmol/L)	15.9	< 26.0
Aspartate aminotransferase (U/L)	19	15–40
Lactate dehydrogenase (U/L)	233	120–250
Total cholesterol (mmol/L)	1.15	3.0–5.7
Triglycerides (mmol/L)	1.8	< 1.7
Albumin (g/L)	38.6	35.0–52.0
Globulin (g/L)	36.1	15.0–30.0
A/G	1.10	1.2–2.4
Fibrinogen (g/L)	4.02	2.0–4.0
Ferritin (μg/L)	1060.6	22.0–322.0
Erythrocyte sedimentation rate (mm/h)	26	< 15
IgG (g/L)	17.53	8.60–17.40
IgA (g/L)	2.53	1.0–4.2
IgM (g/L)	0.55	0.3–2.2
NK cell count (CD16+, CD56+, cells/μL)	110.57	127–987
NK cell count (CD16+, CD56+, %)	10.18	6.7–30.9
T cell count (CD3+, cells/μL)	883.61	797–2370
T cell count (CD3+, %)	81.32	53.7–80.9
B cell count (CD19+, cells/μL)	75.72	86–594
B cell count (CD19+, %)	6.97	5.1–20.3
CD4:CD8 ratio	1.83	0.71–2.78
IL‐6 (pg/mL)	153.6	0.0–20.0
IL‐10 (pg/mL)	6984.8	0.0–5.9
Soluble CD‐25 (pg/mL)	28,688	0–6400
HBsAg	Negative	
HCVAb	Negative	
TPHA	Negative	
HIV (Ag/Ab)	Negative	
Flu A‐Ag	Negative	
Flu B‐Ag	Negative	
2019‐ncov‐Ag	Negative	

*Note:* HBsAg: hepatitis B surface antigen, HCVAb: hepatitis C virus antibody, Flu A‐Ag: influenza A virus antigen, Flu B‐Ag: influenza B virus antigen.

Abbreviations: HIV, human immunodeficiency virus; TPHA, Trepomema Pallidum Hemagglutination Assay.

**Figure 2:**
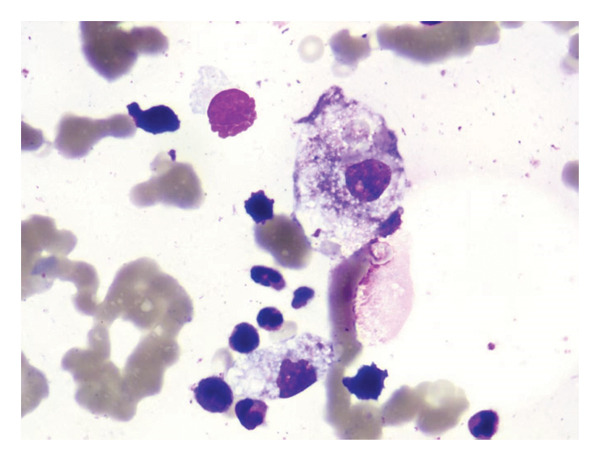
Bone marrow cytology of the patient. Hypercellular marrow with active hematopoiesis. The myeloid‐to‐erythroid ratio is normal. A portion of neutrophils show increased granulation. Erythropoiesis is predominantly at the midlate stages. No increase in blasts or significant dysplastic changes are observed. The megakaryocyte count is normal, but the proportion of platelet‐producing forms is decreased; platelet clumping is rare on the smear. Lymphocytes are not increased and appear morphologically unremarkable. There is a mild increase in mature‐appearing plasma cells. Macrophages are readily seen, with hemophagocytosis observed.

Prior to admission to our institution, all bacteriological tests were negative and broad‐spectrum antibiotics were used in other two hospital. Since the antibiotics had no effect on the fever, we initiated plasma mNGS to detect microbial cell‐free DNA/RNA, enabling comprehensive pathogen genome profiling. The mNGS yielded a positive result for HHV‐8 (Supporting Table S1). Then, acyclovir was added immediately. However, the condition of the patient deteriorated with worsening pancytopenia, persistent high fever, decreased consciousness, hypercalcitoninemia, and acute renal failure. Two days later, bone marrow cytology revealed a hypercellular marrow with plasmacytosis and hemophagocytosis (Figure 2). No schistocytes were observed on the peripheral blood smear. A diagnosis of HLH was presumed and methylprednisolone 40‐mg qd was added. Then, soluble CD25 was measured. Three days later, soluble CD25 level was 28,688 pg/mL and a repeat triglyceride level was 3.55 mmol/L (normal range below 1.7 mmol/L). We advised the patient to use Etoposide (VP‐16), but the patient’s relatives refused. During this phase, the patient achieved temperature stability alongside steadily declining inflammatory markers (procalcitonin and C‐reactive protein [CRP]). Renal function showed significant recovery, paralleled by a gradual rise in platelet counts toward normal levels. Due to financial reasons, the patient’s family opted to discharge the patient and return to their hometown for further treatment at a local hospital. Unfortunately, the patient succumbed to septic shock caused by a severe nosocomial (hospital acquired) bacterial infection at a local hospital 3 months later.

## 3. Discussion

HLH is a critical and potentially fatal syndrome marked by uncontrolled hyperinflammatory condition, which can rapidly progress to multiple organ dysfunction and mortality without immediate, targeted therapeutic intervention. HLH syndrome is typically stratified into two distinct categories: primary HLH characterized by Mendelian inheritance patterns and secondary HLH associated with nongenetic triggers such as infections, malignancies, or autoimmune disorders [1]. The most frequent trigger of secondary HLH is infections (49.9%) in ICU, with subsequent frequencies attributable to malignancies (28.0%) and autoimmune diseases (12.1%) [5]. The etiological spectrum of infection‐induced HLH is predominantly driven by Epstein–Barr virus (EBV) and cytomegalovirus (CMV), with minority cases attributable to alternative pathogens including HIV, varicella zoster virus (VZV), and influenza viruses [2, 5]. COVID‐19 [6], tuberculosis [7], dengue [8], and babesiosis [9] were also reported to be the triggers of secondary HLH in a few case reports. Sporadic case reports have documented HHV‐8‐associated HLH, but these cases demonstrate concurrent HIV coinfection or involve transplant recipients [10, 11]. In our case, the patient appeared HHV‐8‐associated HLH without HIV infection or transplantion.

The diagnosis of HLH remains challenging due to the lack of specific diagnostic assays, especially secondary HLH. The HLH‐2004 criteria are commonly used in clinical practice. According to these criteria, the diagnosis of HLH requires fulfillment of any five of the following eight criteria: fever, splenomegaly, bicytopenia (hemoglobin, platelets, and neutrophils), hypertriglyceridemia or hypofibrinogenemia, hemophagocytosis, hyperferritinemia, low or absent NK‐cell activity, and elevated sCD25 levels [1]. The updated revision presents two independent diagnostic strategies (a cellular based on cytotoxicity and a genetic pathway), while designating the fulfillment of five out of the remaining seven criteria (excluding NK‐cell activity) as the third diagnostic pathway [12]. However, we have encountered several suspected HLH cases where patients rapidly developed multiple organ dysfunction syndrome (MODS) due to either delayed treatment from misdiagnosis during interhospital transfers or inherently rapid disease progression, leaving insufficient time for definitive diagnosis. We, therefore, strive to minimize diagnostic delays by implementing the following protocols for patients presenting with fever and bicytopenia: ferritin testing, peripheral blood smears, and pathogenesis evaluations (including pathogen screening via mNGS, autoimmune markers, immune cell counts, and cytokine profiling) are completed on the day of admission, followed by expedited bone marrow biopsy for suspected cases. Simultaneously, we optimize laboratory turnaround times—as demonstrated in the case reports, mNGS results are typically available within 24 h and bone marrow cytology findings within 72 h—to race against time for life‐saving interventions.

The pathophysiological mechanisms of secondary HLH remain incompletely understood. Several research studies suggest that various pathogenic factors combine and reach a certain threshold, ultimately leading to uncontrolled inflammation and causing fulminant HLH [1, 13]. In our case, HHV‐8 infection was the major trigger of secondary HLH. Previous research has discovered that HHV‐8 expresses E3 ubiquitin ligases involved in immune evasion [14, 15]. In addition, it has been reported that E3 ubiquitin ligases may be involved in the pathogenesis of secondary HLH [16]. Therefore, E3 ubiquitin ligases may be a critical factor in the pathogenesis of HHV‐8‐associated secondary HLH, like the case in our report. HHV‐8 is also reported to be the cause of KICS. KICS is a newly described condition that typically manifests with fever, anemia, thrombocytopenia, hypoalbuminemia, hyponatremia, hepatosplenomegaly, elevated CRP, and increased IL‐6 and IL‐10 levels, along with a high HHV‐8 viral load [4, 17]. The diagnosis of KICS requires meeting clinical and laboratory manifestation criteria along with evidence of KSHV viral activity, while necessitating the exclusion of other disease entities to establish the diagnosis [4, 18]. Our case demonstrates alignment with the majority of KICS diagnostic criteria, suggesting potential overlapping pathogenic pathways between HHV‐8‐associated HLH and KICS pathogenesis. Concurrently, Mario Luppi et al. [19] have emphasized the necessity of differentiating KICS from HLH in specific clinical scenarios, as part of the diagnostic deliberation process. We strongly concur with the perspective. Through review of published KICS case reports [4, 17, 18], we observed that a substantial proportion lacked sCD25 level assessments. This diagnostic gap suggests that a subset of reported KICS cases may represent misclassified HLH entities.

The prognosis of HLH is poor, with an overall mortality rate ranging from 41% to 57.8% and up to 80% in the critically ill [5, 20]. Without appropriate treatment, the median survival time is limited to 1‐2 months, while historical data demonstrate a dismal 5‐year survival rate of less than 5% [21]. HLH is relatively rare, and the diagnosis is often delayed, which is part of the reasons for the high mortality. In the management of secondary HLH, therapeutic intervention targeting both the underlying primary condition and the trigger of HLH (including infections, malignancies, and autoimmune/autoinflammatory disorders) is also important. The clinical emergence of HHV‐8 infection exhibits significant predilection for immunodeficient hosts, particularly within HIV seropositive cohorts and post‐transplantation populations, wherein the deranged immune landscape portends accelerated disease progression and diminished survival outcomes. Through our case report, we seek to advance clinical recognition of HHV‐8‐associated HLH, optimize diagnostic precision, and ultimately improve survival outcomes for patients.

## 4. Conclusions

HLH is a critical and potentially fatal syndrome marked by uncontrolled hyperinflammatory condition. The development of HLH secondary to HHV‐8 infection is seldom observed in clinical practice, particularly in patients with neither transplantation history nor underlying HIV immunodeficiency. Ferritin testing, peripheral blood smears, and pathogenesis evaluations should be immediately implemented for patients presenting with fever and bicytopenia. There might be potential overlapping pathogenic pathways between HHV‐8‐associated HLH and KICS pathogenesis. In addition, E3 ubiquitin ligases may be a critical factor in the pathogenesis of HHV‐8‐associated secondary HLH. But more research studies are needed into its causes.

**Nomenclature**
HLH: Hemophagocytic lymphohistiocytosis
HHV‐8: Human herpesvirus 8
KSHV: Kaposi sarcoma–associated herpesvirus
HIV: Human immunodeficiency virus
PEL: Primary effusion lymphoma
MCD: Multicentric Castleman disease
KICS: Kaposi sarcoma inflammatory cytokine syndrome
mNGS: Metagenomic next‐generation sequencing
CRP: C‐reactive protein
EBV: Epstein–Barr virus
CMV: Cytomegalovirus
VZV: Varicella zoster virus
ICU: Intensive care unit
MODS: Multiple organ dysfunction syndrome
COVID‐19: Coronavirus disease 2019

## Ethics Statement

The patient’s immediate relative, who was authorized by the patient, gave consent for this case report. Informed consent was obtained from the individual(s) for the publication of any potentially identifiable images or data included in this article.

## Conflicts of Interest

The authors declare no conflicts of interest.

## Funding

This work was supported by grants from the National Natural Science Foundation of China (nos. 82001587 and 82241017).

## Supporting Information

Supporting Table S1: Results of the plasma mNGS.

## Supporting information


**Supporting Information** Additional supporting information can be found online in the Supporting Information section.

## Data Availability

The data that support the findings of this study are available in the supporting information of this article.
