# Supplementary material for: HHV‐8‐Associated Hemophagocytic Lymphohistiocytosis in a HIV‐Negative and Nontransplant Man: A Case Report and Literature Review
Source: Case Rep Infect Dis. 2025 Dec 19;2025:9882834. doi: 10.1155/crdi/9882834 (PMC12717441; doi:10.1155/crdi/9882834)
Supplement: Supplementary file 1 — Supporting Information Additional supporting information can be found online in the Supporting Information section. [file CRDI-2025-9882834-s001.docx]

| Table S1 Results of the plasma mNGS | | |
| --- | --- | --- |
|  | Read count | relative abundance(%) |
| Bacteria | None | |
| Mycobacteria | None | |
| Mycoplasma/Chlamydia/Rickettsia | None | |
| Fungi | None | |
| DNA viruses |  |  |
| Human gammaherpesvirus 8  (Kaposi's sarcoma-associated herpesvirus) | 1770 | 84.46 |
| Human betaherpesvirus 5  (cytomegalovirus) | 8 | 0.39 |
| Human gammaherpesvirus 4  (Epstein-Barr virus) | 2 | 0.11 |
| RNA viruses | None | |
| Parasites | None | |
